# Gene Polymorphisms of the Renin-Angiotensin-Aldosterone System as Risk Factors for the Development of In-Stent Restenosis in Patients with Stable Coronary Artery Disease

**DOI:** 10.3390/biom11050763

**Published:** 2021-05-20

**Authors:** Madina Azova, Kalima Timizheva, Amira Ait Aissa, Mikhail Blagonravov, Olga Gigani, Anna Aghajanyan, Leyla Tskhovrebova

**Affiliations:** 1 Institute of Medicine, Peoples’ Friendship University of Russia (RUDN University), 6 Miklukho-Maklaya St, 117198 Moscow, Russia; bogatyreva-kb@rudn.ru (K.T.); ait-aissa-a@rudn.ru (A.A.A.); blagonravov-ml@rudn.ru (M.B.); gigani-oo@rudn.ru (O.G.); agadzhanyan-av@rudn.ru (A.A.); tskhovrebova-lv@rudn.ru (L.T.); 2 National Medical Research Center of Cardiology of the Ministry of Healthcare of the Russian Federation, 15a 3rd Cherepkovskaya St, 121552 Moscow, Russia; 3 Federal Research and Clinical Center of Physical-Chemical Medicine of Federal Medical Biological Agency, 1a Malaya Pirogovskaya St, 119435 Moscow, Russia

**Keywords:** percutaneous coronary interventions, in-stent restenosis, gene polymorphisms, renin-angiotensin-aldosterone system

## Abstract

This study investigated the renin-angiotensin-aldosterone system (RAAS) gene polymorphisms as possible genetic risk factors for the restenosis development in patients with drug-eluting stents. 113 participants had coronary artery disease and underwent stenting. The control group consisted of 62 individuals with intact coronary arteries. Patients were divided into two groups: with in-stent restenosis (ISR) and without it. The patients with ISR were classified into subgroups by the terms of the restenosis development and age. Real-time PCR and Restriction Fragment Length Polymorphism-PCR were used to genotype the study participants for RAAS gene polymorphisms. We found that the development of restenosis is generally associated with the minor A allele for renin (REN) rs2368564 and the major TT genotype for angiotensinogen (AGT) rs699. The heterozygous genotype for AGT rs4762 acts as a protective marker. A minor A allele for angiotensin II type 2 receptor (AGTR2) rs1403543 is associated with a risk of restenosis in people under 65 years old. Among patients with the early ISR, heterozygotes for angiotensin II type 1 receptor (AGTR1) rs5186 are more frequent, as well as A allele carriers for AGTR2 rs1403543. A minor homozygous genotype for REN rs41317140 and heterozygous genotype for aldosterone synthase (CYP11B2) rs1799998 are predisposed to the late restenosis. Thus, to choose the effective treatment tactics for patients with coronary artery disease, it is necessary to genotype patients for the RAAS polymorphisms, which, along with age and clinical characteristics, will allow a comprehensive assessment of the risk of the restenosis development after stenting.

## 1. Introduction

One of the most common medical approaches to the treatment of coronary artery disease (CAD) is the percutaneous coronary intervention (PCI) which became frequent due to high efficiency and safety of this procedure. Modern-day advances in pharmacotherapy and the device innovations over the last thirty years enhanced the benign outcomes of patients with unstable or multivessel CAD, and multiple co-morbidities, treated by PCI [1]. The advent of drug-eluting stents (DES) has revolutionized endovascular surgery, reducing the frequency of in-stent restenosis (ISR) from 20–40% to 6–12% [2]. However, the sharp rise in the total number of DES placements (e.g., more than one million procedures performed annually in the U.S.) has resulted in an increase in the absolute number of ISR cases. Despite technological advances, restenosis remains the main limitation of interventional cardiology, leading to the need for re-PCI in the intervened segment and increasing the global stent market charge numbering in billions of dollars [3]. Identification of risk factors and mechanisms underlying ISR is necessary for understanding the process, the risk stratification, and optimal treatment development. Restenosis, as a physiological response to mechanical damage, involves two mechanisms which are neointimal hyperplasia and vessel remodeling [4]. Several factors such as age, diabetes mellitus, hypertension, stenting of small coronary arteries, and final total length of stents have been shown to be associated with an elevated risk of restenosis. However, these factors could not explain all cases of ISR development after stent implantation [5], which makes it possible to suspect the involvement of genetic factors. At present, the role of renin-angiotensin-aldosterone system (RAAS) in the production of pro-inflammatory cytokines, proliferation, and vasoconstriction, which can contribute to the initiation and progression of neointimal hyperplasia, has already been established [6,7,8]. The effect of renin and angiotensin II on the migration and proliferation of smooth muscle cells has also been proven [9,10]. In recent decades, researchers from different countries have been actively studying the relationship between components of RAAS and their genes with the development of cardiovascular pathology in general, and in particular, ISR. The lack of consistency in the literature data may be due to various factors, including the genetic structure of different ethnic groups. Therefore, we conducted this study to establish the genetic variants of the RAAS components contributing to restenosis after the PCI in patients with stable CAD.

## 2. Materials and Methods

175 Russian individuals were enrolled to the study, including 113 patients of both sexes (94 men and 19 women) with stable CAD who had previously undergone balloon angioplasty with stenting using drug-eluting stents. A mean age was 56.0 ± 10.7 years. According to the results of the control angiography, the patients were divided into two groups: patients with ISR (n = 54) and patients without restenosis (n = 59). The patients with ISR were classified into subgroups by the terms of the restenosis development (before 12 months, n = 22; after 12 months, n = 32) and age (under 65 years, n = 36; over 65 years, n = 18). The control group consisted of 62 patients with intact coronary arteries according to the results of coronary angiography (53 men and 9 women). A mean age was 49.7 ± 10.8 years. Exclusion criteria were as follows: unstable angina, acute myocardial infarction within the last month, decompensated heart failure, oncological diseases, renal and hepatic failure, treatment with angiotensin-converting enzyme inhibitors or angiotensin II receptor blockers. All subjects gave their informed consent for inclusion before they participated in the study. The study was conducted in accordance with the Declaration of Helsinki, and the protocol was approved by the Local Ethics Committee of the Institute of Medicine of RUDN University (No. 1, 20.09.2018).

The integrated analysis included a study of clinical characteristics, traditional risk factors of CAD (arterial hypertension, dyslipidemia, diabetes mellitus, obesity, smoking history), and echocardiography. We performed repeated coronary angiography in a year after discharge or earlier, in case of return of angina symptoms or positive cardiac tests. Follow-up of angiography allowed the analysis of minimal lumen diameter before and after stenting, length of the lesion, and generation of the implanted stent. Restenosis was identified by symptoms or signs of ischemia (electrocardiographic evaluation or exercise testing) and confirmed by the presence of angiographic stenosis in the stented segment. All patients had been followed up for at least 24 months. The primary endpoint of the study was angiographic restenosis (stenosis of more than 50% of the target vessel). Secondary study endpoints were cardiac death, stent thrombosis, target vessel-related myocardial infarction, and clinically driven target vessel revascularization during the follow-up. Angiographic, clinical, laboratory data of patients were evaluated, as well as genotyping performed on gene polymorphisms involved in pathophysiological mechanisms of endothelial dysfunction and ISR.

The study included polymorphic loci of genes encoding RAAS components: aldosterone synthase (CYP11B2), renin (REN), angiotensin-converting enzyme (ACE), angiotensinogen (AGT), angiotensin II type 1 receptors (AGTR1) and type 2 receptors (AGTR2). Functionally significant loci situated in the coding or regulatory regions of the analyzed genes were preferably selected for the analysis. Genomic DNA was extracted from the peripheral blood. Genotyping for AGT rs699, AGT rs4762, AGTR1 rs5186, AGTR2 rs1403543, ACE rs4646994, CYP11B2 rs1799998, and REN rs2368564 polymorphisms was performed using the real time-PCR method with utilizing commercially available kits (Syntol, Moscow, Russia). To identify a polymorphic variant of REN rs41317140 polymorphism, we used Restriction Fragment Length Polymorphism-Polymerase Chain Reaction (RFLP-PCR). Amplification conditions were as follows: initial denaturation at 95 °C for 5 min succeeded by 30 cycles (denaturation at 95 °C for 45 s, annealing at 60 °C for 30 s, and elongation at 72 °C for 1 min and 30 s). Final elongation was performed at 72 °C for 10 min. Primers: F5′-GCTGTCTTCTGGTGGTACTGCC-3′, R5′-TGCTGGCCATGAACTGGTTCTAGC-3′ [11]. The obtained PCR product (964 bp) was fragmented by restriction enzyme TaqI (SibEnzyme, Novosibirsk, Russia) and separated by 2% agarose gel electrophoresis. Obtained fragments for T allele were 570 and 394 bp, for C allele—964 bp.

The data were analyzed using R-language and SPSS version 20 statistical software. The Chi-square test and Fisher’s exact test were used to compare genotype and allele frequencies between the analyzed groups. Differences were considered as significant at *p* < 0.05. The odds ratio (OR) and 95% confidence interval (CI) were calculated.

## 3. Results

### 3.1. Comparative Clinical Characterization of Groups of Patients with CAD

Analysis of risk factors for CAD, which may predispose to re-narrowing of the vessel after stenting, was carried out. For most of the factors, no significant differences were found between the groups with ISR and without it (Table 1). Significant differences were found for three parameters, which were the occurrence of diabetes mellitus, the frequencies of multifocal atherosclerosis, and multivessel coronary artery disease.

### 3.2. Post-Procedural Medications

According to clinical recommendations, all patients with CAD after PCI took double anti-aggregate therapy, including aspirin (100 mg/day) and clopidogrel (75 mg/day). After 12 months of treatment with clopidogrel and aspirin, all the patients continued receiving aspirin. A dose-adjusted hypolipidemic therapy (atorvastatin or rosuvastatin) was administered to all to achieve the target low-density lipoprotein cholesterol level. Beta-blockers were taken by 60% of patients with ISR and 47% of patients without it (*p* > 0.05).

### 3.3. Comparative Angiographic Characterization of Groups of Patients with CAD

The degree of vessel lesion was comparable in both groups before primary PCI. A multivessel coronary lesion was detected in 68% of patients with ISR and 47% of patients without it (*p* > 0.05). Total occlusions were revealed in 23% of patients in the first group and 22% of patients in the second group (*p* > 0.05). The length of the implanted stents averaged 27.6 mm and 28.3 mm, respectively (*p* > 0.05). The minimum diameter of the stents was 3.1 mm and 2.9 mm, respectively (*p* > 0.05). All the patients were implanted with DES (everolimus, sirolimus, biolimus, paclitaxel) of I, II, and III generations. The ratio of DES of different generations was comparable in the groups (Table 1).

### 3.4. Allelic and Genotypic Frequencies

Table 2 shows the results obtained after genotyping of patients.

The distribution of genotypes for AGT rs699 in the group without restenosis corresponds to that in the control group and significantly differs from the distribution in the group with ISR (*p* = 0.017). Moreover, the frequency of the C allele tends to decrease among patients with ISR (*p* = 0.059) as well as the frequency of homo- and heterozygous carriers of this allele. A reduced frequency of the heterozygous genotype among patients with restenosis was also detected for the rs4762 polymorphism (*p* = 0.085) with a significantly lower incidence among those with restenosis developed later than 12 months after stenting. At the same time, an increased frequency of minor TT homozygotes was revealed in the latter subgroup (*p* = 0.007).

The genotypic distribution for AGTR1 rs5186 polymorphism was significantly different in the subgroup of patients with early restenosis (*p* = 0.031). There was an evident predominance of AC heterozygotes.

Genotyping of AGTR2 rs1403543 polymorphism was carried out only for males, since the gene is located on the X-chromosome, and regarding the process of lionization, it is not possible to evaluate the results unequivocally in women with a heterozygous genotype. In men with CAD, the hemizygotes for the A allele were significantly more frequent than in the control group (*p* = 0.047). An interesting fact is that this allele is more prevalent in patients younger than 65 years, and those with the early restenosis, although the differences from the group without ISR do not reach a statistically significant level (*p* = 0.055 and 0.078, respectively).

Analysis of genotype and allele frequencies for I/D polymorphism of the ACE gene showed a significantly higher occurrence of the DD genotype in the group of patients with CAD (*p* = 0.031) compared with the control.

As a part of our study, it was found that frequencies of REN rs2368564 alleles and genotypes in the group of patients with restenosis were significantly different from those in patients without ISR (*p* = 0.002 and 0.0004, respectively), which suggests the association of the minor AA genotype with the restenosis development. Moreover, it was found that these indices are highest among patients with restenosis older than 65 years (*p* < 0.0001). A study of the genotype distribution for polymorphic locus REN rs41317140 revealed a significant difference between the control group and the patients with CAD (*p* = 0.0002). Minor homozygotes were detected only in the patients with CAD, and more frequently among the patients with late restenosis (*p* = 0.03).

Analysis of CYP11B2 rs1799998 polymorphism data showed no association with restenosis in general. However, completely different genotypic distributions in subgroups divided by the term of the restenosis development (*p* < 0.0001) were notable. Among the patients with late restenosis, the heterozygotes predominate, so they also significantly differ from the subgroup without ISR (*p* = 0.0005).

## 4. Discussion

ISR has been described as a complex process involving a large number of clinical, biochemical, and genetic factors affecting endothelial function. There are also no clear diagnostic criteria for the ISR risk stratification. The preventive tendency of modern medicine shows the necessity for the search for ISR risk factors not only after PCI but also before it to use the optimal personalized tactics in the treatment of CAD. Clinical risk factors of ISR include diabetes mellitus, arterial hypertension, dyslipidemia, and smoking. Angiographic parameters associated with restenosis are the minimal stent diameter and maximal stent length.

Analysis of clinical and angiographic characteristics showed that the patients in the groups with ISR or without it are comparable in the clinical, laboratory, and angiographic parameters. We found the association of ISR with type 2 diabetes mellitus and multivessel atherosclerosis, which coincided with current knowledge.

The main pathophysiological mechanism for the development of restenosis is neointimal hyperplasia caused by the smooth muscle cells’ proliferation and extracellular matrix production. Chronic activation of RAAS leads to fibrosis, inflammatory response, and hypertrophy, which causes myocardial and vessel wall remodeling, and cardiovascular dysfunction.

Renin is synthesized as preprorenin in juxtaglomerular kidney cells, which is then converted into prerenin and released or changed to the active renin. The active renin formation is a controlled process stimulated by low blood pressure, hypovolemia, and other factors, while angiotensinogen is constantly released from the liver. Renin cleaves angiotensinogen and converts it into inactive angiotensin I. Angiotensin converting enzyme (ACE), which is synthesized in endothelial cells, converts angiotensin I into angiotensin II acting via the two types of receptors: AGTR1 and AGTR2. Interaction with AGTR1 leads to sodium retention, vasoconstriction, and the release of aldosterone from the adrenal glands, which, in turn, acts as a key regulator of water and salt balance. Angiotensin increases the expression of the aldosterone synthase gene and inhibits the renin secretion by negative feedback. The effects of angiotensin II mediated by type 2 receptors are generally opposite to those associated with type 1 receptors. Angiotensin and aldosterone are also produced in the brain, kidneys, blood vessels, and heart. Local RAAS components play an important role in the normal functioning and remodeling of the cardiovascular system. They are modulated by mechanical distension of the myocardium and blood vessels, reactive oxygen species, and inflammation. The ACE-independent formation of AGT II is possible in tissues via chymase released from mast cells, cardiac fibroblasts, and vascular endothelial cells during acute and chronic tissue damage and remodeling [12,13,14].

At present, the association of polymorphisms of genes encoding key components of the RAAS, with the development of such diseases as hypertension, acute myocardial infarction, coronary heart disease, and atherosclerosis has been found in different populations [15,16,17]. Several studies have shown correlation of a number of polymorphisms with the restenosis after balloon angioplasty and bare metal stents implantation [18,19,20,21]. However, information about the involvement of different gene polymorphisms for the ISR development in patients with DES is contradictory.

Among the common AGT gene polymorphic variants, M235T (rs699) and T174M (rs4762) are most extensively studied. CC homozygotes for the rs699 polymorphic locus have a 10–20% increased plasma AGT level, which is probably due to the linkage disequilibrium with the G-6A locus in the promoter region of that gene [22,23]. The literature data on the association of AGT gene polymorphisms with the development of restenosis is controversial. Several studies have not revealed the presence of the association [8,24,25,26,27]. Our results regarding rs699 showed no association with a minor allele. In the group with restenosis, the frequency of major TT homozygotes was increased (OR 2.362; CI 1.111–5.020), especially in the subgroup of patients younger than 65 years old (OR 2.585; CI 1.221–5.471). Thus, we can suggest that an increased level of angiotensinogen is not involved in the ISR development. A decreased frequency of the heterozygous genotype among patients with restenosis was also observed for the rs4762 polymorphism (OR 0.396; CI 0.170–0.430). The lowest incidence was in individuals with late restenosis (OR 0.255; CI 0.098–0.667), which makes it possible to consider it as a protective marker against restenosis development.

The ACE rs4646994 polymorphic locus of the angiotensin-converting enzyme gene has been studied for many years, and its association with cardiovascular diseases is well known. It was shown that individuals with the DD and ID genotypes have increased the plasma ACE level compared with homozygotes II. A number of researchers have shown the association of the DD genotype with restenosis [8,24,28,29], although this fact is not confirmed in some studies [27]. Analysis of our data showed association of the DD genotype with coronary artery disease in general, but not with restenosis.

AGTR1 rs5186 polymorphism is one of the most studied loci of the AGTR1 gene. It has been established that the C allele is associated with higher gene expression, which is probably related to the post-transcriptional regulation of gene activity via miRNAs [30]. Information regarding the association of this polymorphism with ISR is contradictory [20,27]. We found a marked increase in the frequency of heterozygotes in a subgroup of patients with early restenosis (OR 2.121; CI 1.196–3.775). According to the study conducted by Zhu M., et al. [24], the AC genotype is a risk factor for individuals over 60 years of age. In our study, we indeed observed a slight increase in the frequency of heterozygotes among patients over 65 years of age, but the difference was not statistically significant.

Data on the functional role of the AGTR2 rs1403543 polymorphism are ambiguous. In 1999, it was found that the mentioned locus was located near the intron 1 branch point, and could affect the splicing process [31,32]. However, the study published in 2005 showed the influence of this locus not on splicing, but on the gene expression level. It appeared that the G allele was associated with the more active expression and, accordingly, a large number of receptors. Consequently, it has a protective influence on cardiovascular diseases [33]. At present, regardless of the molecular basis that determines this phenomenon, and conflicting data [34], it is believed that the A allele predisposes to the development of cardiovascular pathology [35,36,37]. The results of our study are entirely consistent with this concept: hemizygotes for the A allele are significantly more frequent among men with coronary artery disease than in the control group (OR 1.833; CI 1.046–3.214). An interesting fact is that the frequency of this allele is particularly high in subgroups of patients younger than 65 years old (OR 1.847; CI 1.028–3.318) and with the early restenosis (OR 1.832; CI 1.024–3.281), although the distribution of alleles in the mentioned subgroups tends to differ compared with the group without ISR, suggesting the need for confirmation of the data on larger samples size.

Thus, the imbalance in the expression of angiotensin II receptors, particularly, a moderate increase in the expression of type 1 receptors and a decrease in the expression of type 2 receptors, probably plays a significant role in the development of restenosis, especially during the first year after PCI.

REN rs2368564 polymorphism situated in the ninth intron of the renin gene has been actively studied for more than 20 years, and its association with arterial hypertension has been established [38,39,40]. Few studies regarding REN rs41317140 (C-4063T) polymorphism have shown that the SNP is located in the promoter region and is considered as a transcription factor-binding site, as well as the minor allele being associated with increased renin activity [11]. The study of the association of these polymorphisms with ISR has not been conducted previously. We found that the frequency of A allele and AA homozygotes for REN rs2368564 polymorphism was significantly higher in the group of patients with ISR, and the minor allele was associated with the development of in-stent restenosis in patients with stable CAD after implantation of the DES (for AA genotype OR 4.062; CI 1.758–9.382). Moreover, it was found that the frequencies were highest among patients with restenosis older than 65 years (for AA genotype OR 8.014; CI 3.487–18.420). The study of the genotype distribution for REN rs41317140 revealed a significant difference between the control group and the patients with coronary artery disease. Homozygotes for the minor T allele were found only in the patients with coronary artery disease, and all the individuals with ISR who had that genotype were patients who developed restenosis later than 12 months after PCI. Similar results were obtained by Afruza R. et al. [11], who studied the association of this polymorphism with arterial hypertension; homozygotes for the minor allele were detected only among hypertensive patients, but not in the control group.

The CYP11B2 rs1799998 polymorphism is located in the promoter region of the aldosterone synthase gene and can affect gene expression. The data regarding its association with cardiovascular pathology are numerous and contradictory [41,42,43]. Ryu SK et al. [8] did not reveal an association of this polymorphism with restenosis. A similar result was obtained in our study. However, the patients with late restenosis had a markedly different genotype distribution with the predominance of heterozygotes (OR 3.019; CI 1.677–5.432).

Thus, the main differences were found for subgroups of patients classified in accordance with age and term of the ISR development, so it would be reasonable to conduct a similar study with a bigger sample size.

## 5. Conclusions

We studied eight polymorphic loci in six genes of the RAAS components. It was found that only minor allele A for rs2368564 polymorphism of the renin gene and major TT genotype for rs699 polymorphism of the angiotensinogen gene were associated with the development of restenosis in general, whereas the heterozygosity for rs4762 of the angiotensinogen gene acted as a protective marker. However, further stratification of patients with ISR by age and term of ISR development revealed the presence of specific genetic predictors. Thus, minor allele A for AGTR2 rs1403543 was associated with the risk of restenosis in patients under 65 years of age. Heterozygotes for the AGTR1 rs5186 polymorphism and carriers of allele A for AGTR2 rs1403543 were more frequent among patients with early restenosis (before 12 months after PCI). A minor homozygous genotype for REN rs41317140 and heterozygous genotype for rs1799998 of the aldosterone synthase gene were predisposed to the late restenosis. Thus, the choice of the treatment tactics for patients with CAD requires genotyping of patients for the studied gene polymorphisms, which, along with age and clinical characteristics, will allow a comprehensive assessment of the risk and possible terms of the restenosis development after stenting.

## Figures and Tables

**Table 1:** General characteristics of studied groups of patients.

	With in-Stent Restenosis (ISR) (n = 54)	without ISR (n = 59)	*p*-Value	Control (n = 62)
Age, years (M ± SD)	60.0 ± 10.1	58.8 ± 8.0	0.885	49.7 ± 10.8
Smoking, %	60	58	0.774	28
Obesity, %	34	27	0.282	21
Dyslipidemia, %	62	52	0.153	14
Diabetes mellitus, %	53 *	8	<0.0001	0
Myocardial infarction, %	36	25	0.091	0
Multifocal atherosclerosis, %	47 *	20	0.0001	0
Arterial hypertension, %	63	56	0.313	52
Systolic blood pressure, mm Hg (M ± SD)	138 ± 7.3	137 ± 7.8	0.952	129 ± 11.2
Diastolic blood pressure, mm Hg (M ± SD)	88 ± 4.9	87 ± 7.8	0.939	79 ± 9.7
Glucose, mmol/L (M ± SD)	4.4 ± 1.0	4.3 ± 1.2	0.973	4.4 ± 0.7
Total cholesterol, mmol/L (M ± SD)	5.2 ± 1.7	4.3 ± 1.8	0.770	4.2 ± 0.8
Low-density lipoproteins, mmol/L (M ± SD)	2.3 ± 1.2	2.4 ± 0.9	0.963	2.4 ± 0.5
Multivessel coronary artery disease, %	68 *	47	0.004	0
Total occlusions, %	23	22	0.866	0
Mean stent length, mm (M ± SD)	27.6 ± 8.1	28.3 ± 8.6	0.925	0
Minimal stent diameter, mm (M ± SD)	3.1 ± 0.54	2.9 ± 0.45	0.935	0
I generation DES (drug-eluting stents), %	20	17	0.585	0
II generation DES, %	66	62	0.659	0
III generation DES, %	14	21	0.264	0

Note: *—significantly different from the group of patients without restenosis (*p* < 0.05).

**Table 2:** Frequencies of alleles and genotypes (%) for polymorphisms of RAAS genes in studied groups.

Gene Polymorphisms	Genotypes and Alleles	Control (n = 62)	CAD (n = 113)	Restenosis (n = 54)	without Restenosis (n = 59)	Restenosis			
						under 65 Years (n = 36)	over 65 Years (n = 18)	before 12 Months (n = 22)	after 12 Months (n = 32)
AGT rs699	TT	31	42	53 *	33	58	44	55	53
	TC	48	36	30 *	42	25	39	32	28
	CC	21	22	17 *	25	17	17	13	19
	T C	55 45	60 40	68 32	54 46	70.5 29.5	63.5 36.5	71 29	67 33
AGT rs4762	TT	14	22	24	20	25	22	14	31 *
	TC	30	15	9	20	8	11	14	6 *
	CC	56	63	67	60	67	67	72	63
	T C	29 71	29.5 70.5	28.5 71.5	30 70	29 71	27.5 72.5	21 79	34 66
AGTR1 rs5186	AA	53	52	47	58	50	39	41 *	50
	AC	27	33	33	32	30	39	50 *	22
	CC	20	15	20	10	20	22	9	28
	A C	66.5 33.5	68.5 31.5	63.5 36.5	74 26	65 35	58.5 41.5	66 34	61 39
AGTR2 rs1403543	A	45	60 **	63	57	71	47	70	56
	G	55	40	37	43	29	53	30	44
REN rs2368564	GG	60	53	39 *	66	44	28 *	36	41
	AG	32	30	37 *	24	36	38 *	36	38
	AA	8	17	24 *	10	20	34 *	28	21
	G A	76 24	68 32	57.5 * 42.5 *	78 22	62 38	47 * 53 *	54 46	60 40
REN rs41317140	CC	45	62 **	56	68	58	50	50	59
	CT	55	32 **	37	29	34	44	50	28 *
	TT	0	6 **	7	3	8	6	0	13 *
	C T	72.5 27.5	78 22	74.5 25.5	82.5 17.5	75 25	72 28	75 25	73 27
CYP11B2 rs1799998	CC	26	26	20	30	22	17	23	19 *
	CT	43	52	60	46	58	61	41	72 *
	TT	31	22	20	24	20	22	36	9 *
	C T	47.5 52.5	52 48	50 50	53 47	51 49	47.5 52.5	43.5 56.5	55 45
ACE rs4646994	II	24	26	26	25	19	39	32	22
	ID	61	45 **	39	51	44	28	32	44
	DD	15	29 **	35	24	37	33	36	34
	I D	54.5 45.5	48.5 51.5	45.5 54.5	50.5 49.5	41 59	53 47	48 52	44 56

Note: *—significantly different from the group of patients without restenosis, **—significantly different from the control group (*p* < 0.05).

## Data Availability

The data that support the findings of this study are not publicly available due to patient confidentiality.
